# Vertebral microstructure marks the emergence of pelagic ichthyosaurs soon after the End Permian Mass Extinction

**DOI:** 10.1038/s41598-025-14335-y

**Published:** 2025-09-05

**Authors:** Lene Liebe Delsett, Mathieu Gabriel Faure-Brac, Victoria Sjøholt Engelschiøn, Alexandra Houssaye, Anusuya Chinsamy, Jørn Harald Hurum, Benjamin P. Kear

**Affiliations:** 1 Natural History Museum, University of Oslo, Sars’ gate 1, Oslo, 0562 Norway; 2 Département Adaptations du vivant, UMR 7179 CNRS/Muséum National d’Histoire Naturelle, 57 rue Cuvier CP-55, Paris, 75005 France; 3 Department of Biological Sciences, University of Cape Town, Private Bag, Rondebosch, 7700 South Africa; 4 Department of Palaeobiology, Swedish Museum of Natural History, Stockholm, 10405 Sweden

**Keywords:** Ichthyopterygia, Ichthyosauria, Triassic, Microanatomy, Histology, Growth, Locomotion, Palaeontology, Animal physiology

## Abstract

Ichthyosaurs were the first fully marine tetrapods, and evolved a streamlined body, flippers, live birth, and endothermy-like physiology. However, the transition to these adaptations and how it relates to divergence into ocean environments is ambiguous. Here, we use vertebral bone microstructure to document the first ontogenetic series of two Early Triassic taxa that include the oldest ichthyosaur foetal fossils. One series is from *Grippia*, an early ichthyopterygian with a small body, and limbs with some plesiomorphic features. The other is a large, contemporaneous ichthyosaur, *Cymbospondylus*. Together, they phylogenetically bracket the ichthyopterygian-ichthyosaurian transition. *Grippia* has a unique microstructure with a distinctive compacted outer layer, whereas *Cymbospondylus* vertebrae are cancellous throughout, indicating a different ecology and swimming style. The dissimilar distribution of woven-parallel complex in the histology between the two taxa indicates that growth progressed at different speeds. We also document birth lines in ichthyosaurs for the first time. Pelagic, tail-propelled, rapid-growing ichthyosaurs were thus present less than five million years after the End Permian mass extinction, alongside more anguilliform ichthyopterygians. These data capture the ecological and evolutionary transition from reptiles with eel-like swimming to whale-like ichthyosaurs, implying a paradigm shift in ecology and physiology that paved the way for ichthyosaur radiation.

**Supplementary Information:**

The online version contains supplementary material available at 10.1038/s41598-025-14335-y.

## Introduction

Only a handful of land-living vertebrates have ever become fully pelagic, and these transitions are rapid in geological time^1,2^. Ichthyosaurs were the first fully marine tetrapods, and evolved a streamlined body, flippers, live birth and endothermy-like body temperatures^3–8^. However, timing and ecology of their transition from land to water and their earliest diversification is poorly understood^9,10^. Fossil discoveries of the last fifteen years indicate either a rapid evolution of pelagic adaptations and larger body size following a land-water transition after the End Permian Mass Extinction (EPME), or an ichthyopterygian origin before the EPME^7,11,12^. Understanding the early evolution of key adaptations for pelagic top predators can illuminate ichthyosaur origins^13^and mapping the timing and sequence of these adaptations will also increase our understanding of the evolution leading to the classical, thunniform taxa of the Jurassic and Cretaceous^14^.

Here, we use vertebral bone microstructure to pinpoint the earliest stages of pelagic adaptations for these top predators. Vertebral centra are common skeletal components in the fossil record, and provide an untapped resource for exploring morphological evolution, and for which development is scarcely studied in an evolutionary context^15,15,17^. Internal bone microstructure records signals of ontogeny, ecology, locomotion and metabolism^18,18,20^ at microanatomical and histological scales. Microanatomical data reveal the inner architecture and compactness of bone, which is associated with locomotion and other physical forces^17,21,22^. Histological data such as vascularization, tissue types and osteocytes, on the other hand, can be used as proxies for physiological processes. Vertebral microstructure has been used to understand aquatic adaptation in various vertebrates^17,19,23,23,25^ yet data is limited, with ichthyopterygians as a key example^26,27^. Ichthyopterygians were one of the most specialized and long lived clades, with all taxa having amphicoelous centra that were unfused to the neural arch^28^ and that were separated by intervertebral disks^29^.

**Fig. 1:**
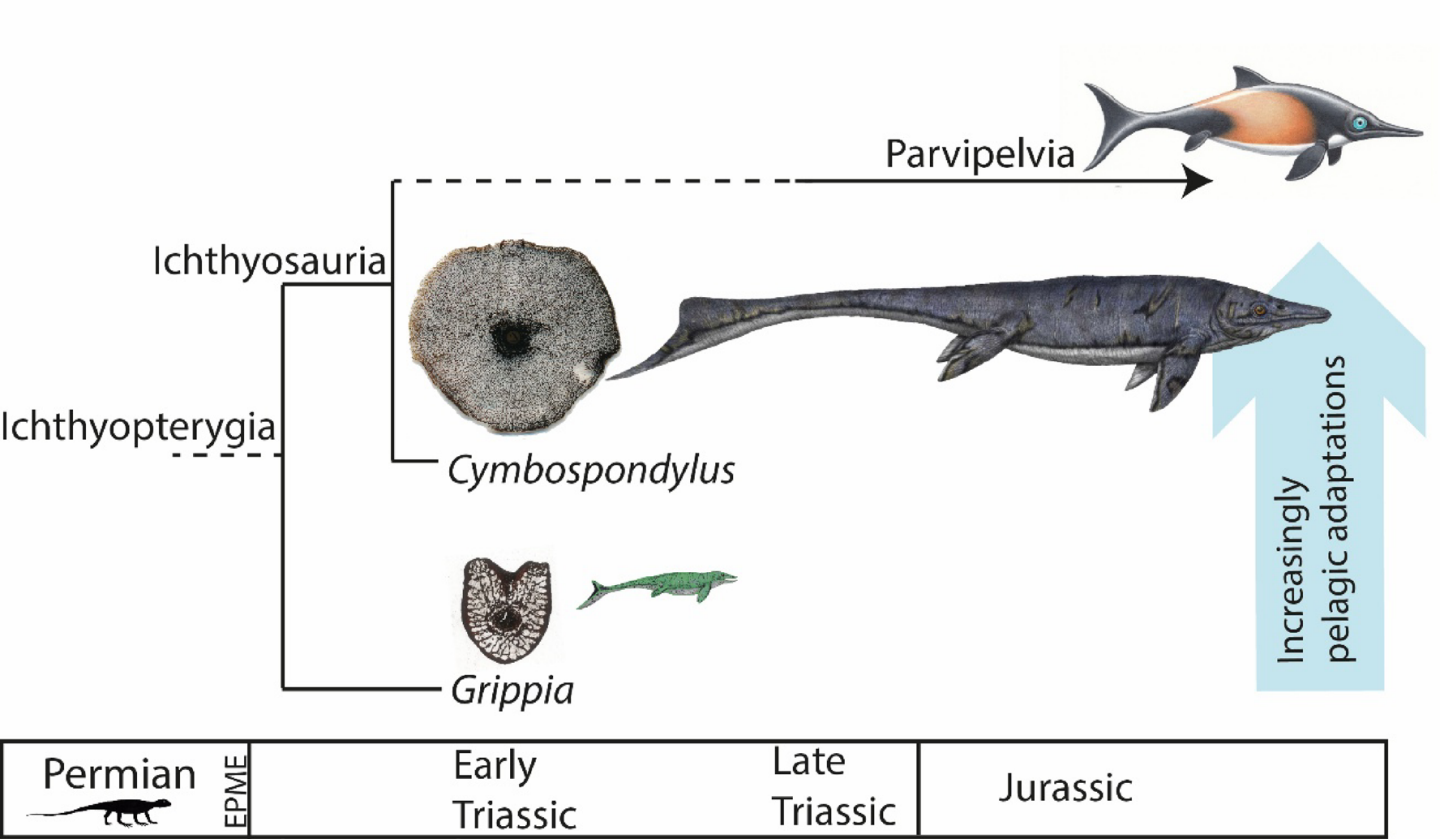
Vertebral microstructure in *Grippia* (PMO 250.499) and *Cymbospondylus* (PMO 230.627) from the Early Triassic point to different locomotion and ecology. EPME End Permian Mass Extinction. Illustrations: Esther van Hulsen, Jakub Kowalski and Piotr Janecki.

**Fig. 2:**
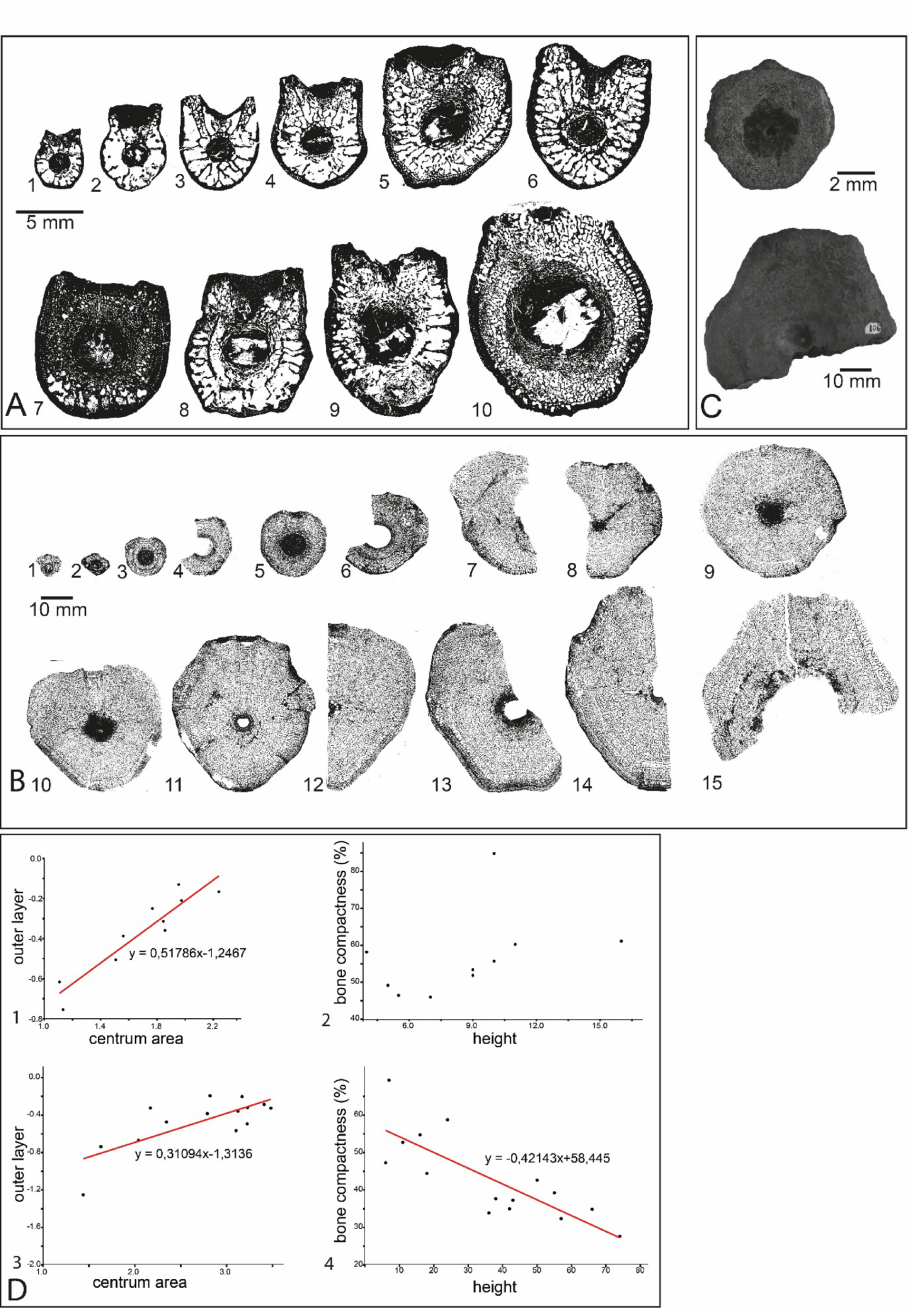
(**A**, **B**) Vertebral microanatomy in transverse section of Early Triassic specimens used in this study. (**A**) *Grippia*. To scale, scale bar = 5 mm. 1: PMO 250.504. 2: PMO 250.503. 3: PMO 250.502. 4: PMO 250.501. 5: PMO 250.500. 6: PMO 250.499. 7: PMO 250.498. 8: PMO 250.497. 9: PMO 250.496. 10: PMO 233.890. (**B**) *Cymbospondylus*. To scale, scale bar = 10 mm. 1: PMO 229.744. 2: PMO 230.686. 3: PMO 231.342. 4: PMO 231.337. 5: PMO 230.738. 6: PMO 230.842. 7: PMO 230.870. 8: PMO 231.220. 9: PMO 230.627. 10: PMO 229.734. 11: PMO 229.743. 12: PMO 229.741. 13: PMO 229.740. 14: PMO 229.735. 15: PMO 229.745. (**C**) *Cymbospondylus* centra: The smallest, foetal (PMO 229.744, top) and the largest, adult (PMO 229.745) with unfinished and finished external bone texture, respectively. (**D**) Quantitative results for microanatomy. 1 (*Grippia*) and 3 (*Cymbospondylus*) shows Mean outer layer (log) versus total area (log); 2 (*Grippia*) and 4 (*Cymbospondylus*) shows Compactness versus dorsoventral height. 1: significant linear relationship (GLM) *p* < 0.01. 2. No significant linear relationship. The outlier is PMO 250.498 (see text). 3: significant linear relationship (GLM) *p* < 0.01. 4: significant linear relationship (GLM) *p* < 0.01.

We compiled comprehensive ontogenetic series of fossil dorsal vertebral centra for two proxy taxa from the Early Triassic, phylogenetically bracketing the origin of Ichthyosauria as a pelagic reptile clade (Figs. 1 and 2A,B)^30^. *Grippia* Wiman, 1928^31,32^ was a small-sized (ca. 1.5 m) early ichthyopterygian^9,33,33,35^ (Fig. 1) with elongated vertebrae, that possibly retained some plesiomorphic characteristics, such as relatively long limb bones^36,36,38^. *Cymbospondylus* Leidy, 1868^39,40^, on the other hand, on the lineage towards the classical dolphin-shape ichthyosaurs, attained whale-sizes, had short vertebrae, an elongated tail and probably a pelagic lifestyle^7,9,34,34,36,39,41^. All the vertebrae in our study sample originate from two different well-documented faunal assemblages at Svalbard (Supplementary material)^39,42^. Together, they chronostratigraphically span the early to late Spathian, with a time frame of approx. two million years, from *Grippia* at approx. 249 Ma, in a transgressive succession of increasing water depth.

We selected specimens (10 *Grippia* and 15 *Cymbospondylus*) from the entire available size range (Supplementary table 1), and because all centra are from the dorsal region, an increase in size is interpreted as an increase in body size and thus assumed to illustrate ontogenetic stage. The same approach was used in previous studies comparing bone microstructure at different ontogenetic stages^14,43^. Using size as an indicator of ontogenetic stages is common, but can be inaccurate^44^and intraspecific and sexual dimorphism may influence the results.

For comparisons with existing literature, and because they are reliably identified based on anatomical criteria, our study focused on bone microstructure of centra from the dorsal region of the two ichthyopterygians, recovered from bonebeds at Spitsbergen (Supplementarytable 1). The material was collected in 2014–2016 from the northern flank of Marmierfjellet in Central Spitsbergen^45^. The *Grippia* specimens were collected from the Grippia bonebed (Early Triassic, Early Spathian), deposited in a mid to distal shelf setting^42^. Ten centra were selected for our study based on the following criteria for assignment to the dorsal region of *Grippia*’s vertebral column: (1) small size (dorsoventral height less than 40 mm)^27,32^(2) amphicoelous^28^(3) with para- and diapophyses^32,34^ although the two are often connected, rather forming an upper and a lower confluent facet and (4) relatively long anteroposterior length compared to other ichthyopterygians^28,32,46^, based on this, centra were selected where height, width and length were approximately equally long.

The *Cymbospondylus* material comes from the Lower Saurian niveau, first described by Wiman (1910) and interpreted as a distal shelf setting^39^. The Lower Saurian niveau is a silty shale horizon rich in isolated, three-dimensionally preserved vertebrate remains. It is in the uppermost section of the Lower Triassic Vendomdalen Member, Vikinghøgda Formation and is of Spathian age^39,45^. Fifteen centra from *Cymbospondylus* were selected based on a truncated diapophysis on the anterior margin of the centrum^39,41,47,48^.

## Results

### Microanatomical and histological features

For both *Grippia* and *Cymbospondylus*, the largest centra have well finished bone surfaces (Fig. 2C), which is a sign of osteological maturity, and we interpret them as adult (sexually mature). The smallest centra are foetal, supported by several lines of evidence: Firstly, their miniature size, which is significantly smaller than the adults of both *Grippia* (the smallest centrum in the sample is 4 mm high, the largest 16 mm, Fig. 2A1–10) and *Cymbospondylus* (smallest centrum is 6 mm, largest is 66 mm, Fig. 2B1–15). In a confirmed case, foetal centra associated with a gravid ichthyosaur were approximately 32% the height of those of the parent^49^ which is far larger than our specimens. Secondly, the smallest *Cymbospondylus* centra have a porous external texture (Fig. 2C), often used as an osteological indicator of early ontogeny^44^. Middle Triassic *Cymbospondylus* foetal vertebrae^27,49^ also share the same microanatomical features. The foetal *Grippia* centra in our series are among the oldest foetal ichthyopterygian material known (Fig. 2A1,2), while those of *Cymbospondylus* are the oldest ichthyosaurian foetal fossils (Fig. 2B1–4). The earliest ichthyopterygian foetuses are from a gravid *Chaohusaurus* (Spathian, approx. 248.8 Ma)^4^. Younger foetal material is known from Middle to Upper Triassic *Besanosaurus*^50^ *Mixosaurus*^51^ and *Shonisaurus*^52^. Early to Middle Jurassic *Stenopterygius*^53,54^ *Leptonectes*^55^ and *Ichthyosaurus*^56,57^ to Cretaceous *Maiaspondylus*^58^ and *Platypterygius*^59^^60^. Thus, our vertebral centra series capture ontogeny from an early foetal stage, through to juvenile to adulthood, providing a unique perspective on ichthyopterygian ontogeny in the early Triassic not covered in previous studies^14,27,43^.

**Fig. 3:**
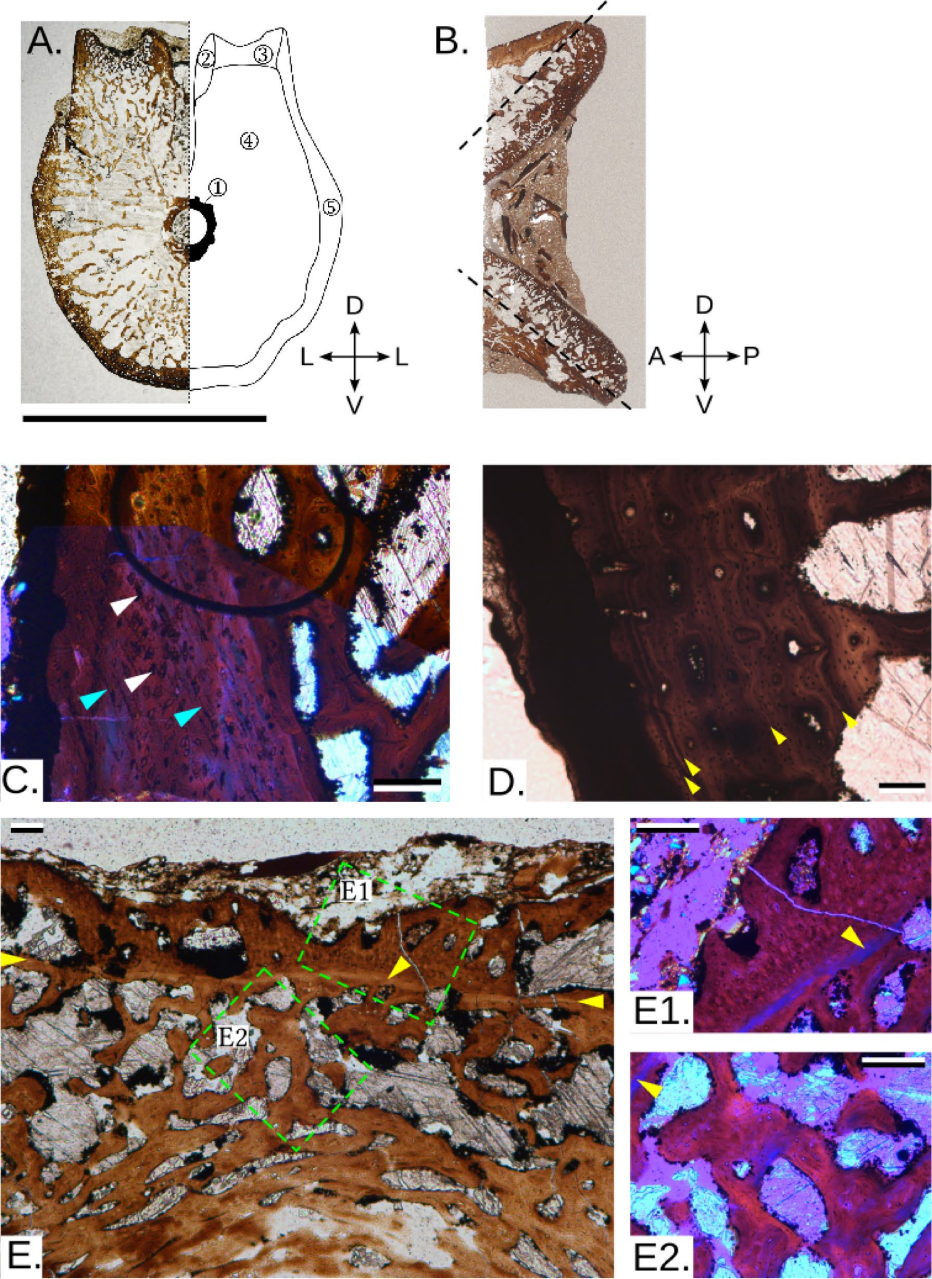
*Grippia*. (**A**) Transverse section of PMO 233.890 with associated symmetrical axes and a schematic representation of the different areas on the right: (1) Notochordal ring, (2) Dorsocentral region, (3) Dorsolateral cone, (4) Inner cancellous periosteal region, (5) Outer bone layer; (**B**) Sagittal section of PMO 250.496 with associated symmetrical axes. Anterior and posterior directions are unknown, and orientation is only indicative. The dashed lines delineate the inner periosteal territories (on the top and bottom) and the outer endochondral territories (in between, on the right); (**C**) Magnification on one of the cones in the transverse section of PMO 233.890, with a mix of LPL and XPL. White arrowheads indicate WB osteocyte lacunae, and blue arrowheads indicate Sharpey’s fibres. The circle is an artefact and does not indicate anything; (**D**) Growth lines in the transverse section of PMO 250.499. Yellow arrowheads indicate LAGs; (**E**) Notochordal ring and dorsocentral area in the transverse section of PMO 250.503, with the birth line indicated with yellow arrowheads. E1 and E2 are magnified pictures of pre- (**E2**) and post- birth (**E1**) histological deposition in XPL. Scale bars --- (A) 1 cm; (B) NA; C., E., E1. and E2. 100 μm; D. 200 μm. *D* dorsal, *V* ventral, *L* lateral, *LPL* linearly polarized light, *A* anterior, *P* posterior, *XPL* cross-polarized light.

**Fig. 4:**
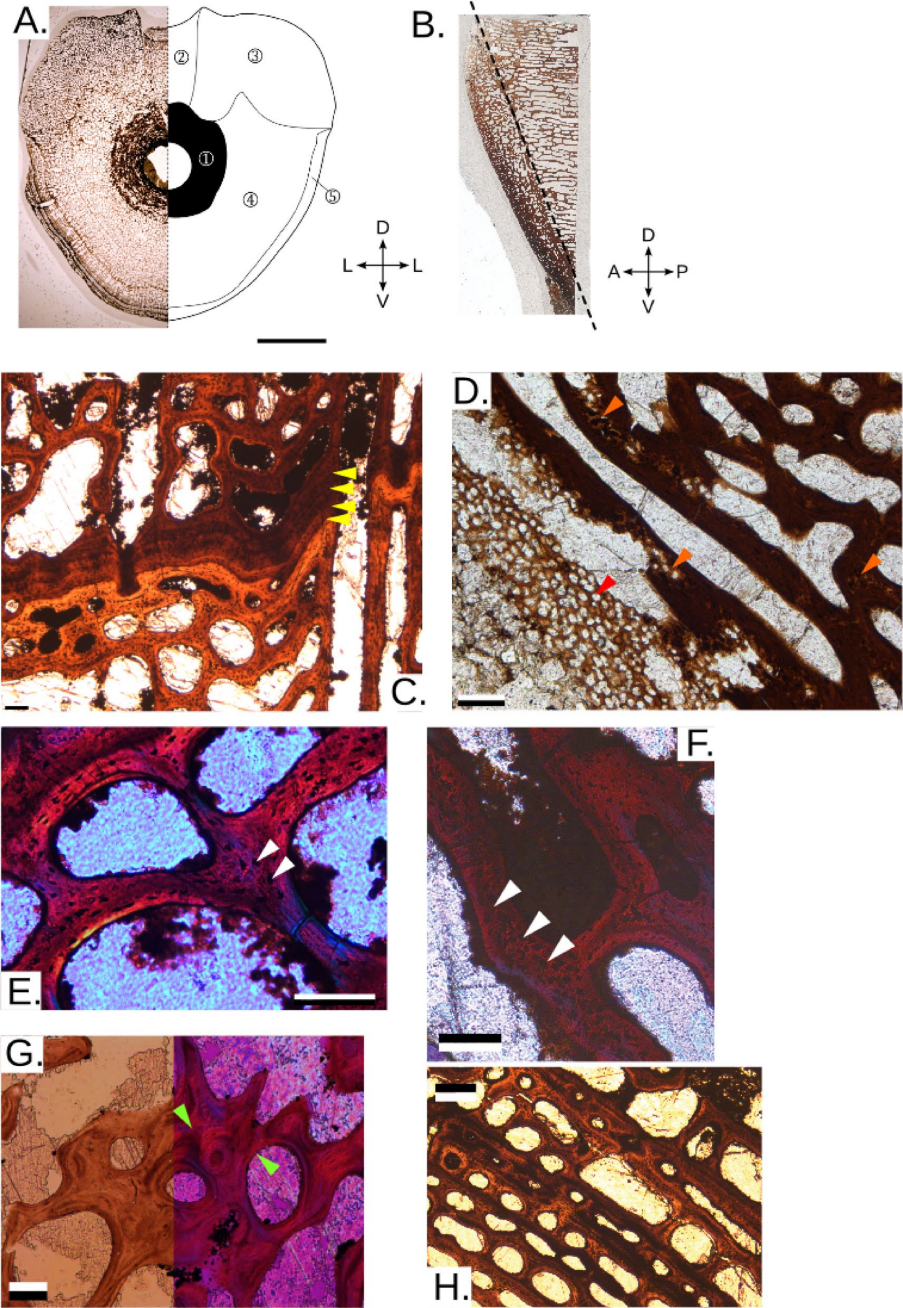
*Cymbospondylus*. (**A**) Transverse section of PMO 229.734 with associated symmetrical axes and a schematic representation of the different areas on the right: (1) Notochordal ring, (2) Dorsocentral region, (3) Dorsolateral cone, (4) Inner cancellous periosteal region, (5) Outer bone layer; (**B**) Sagittal section of the dorsal half of PMO 230.627 with associated symmetrical axes. Anterior and posterior directions are unknown, and orientation is only indicative; (**C**) Growth lines in the transverse section of PMO 229.743. Yellow arrowheads indicate LAGs; (**D**) Inner edge of the notochordal ring in the transverse section of PMO 231.342, displaying calcified cartilage (red arrowhead pointing on large globular lacunae typical of this tissue) and ongoing endochondral ossification (orange arrowheads). (**E**) and (**F**). Woven parallel complex in outer layers in transverse section of PMO 229.743. (**E**) is from trabeculae in the outer layer, (**F**) is from the inner, periosteal region. White arrowheads indicate globular osteocytes, typical of WB. (**G**) Secondary osteon in the notochordal ring in PMO 229.743, with cementing lines. (**H**) Layering in transverse section of PMO 229.734, displaying the regular circumferential deposition progressively being remodeled in new trabeculae. Scale bars --- (A) 1 cm; (B) NA; (C) and H. 200 μm; D., E., F. and G. 100 μm. *D* dorsal, *V* ventral, *L* lateral, *A* anterior, *P* posterior.

Some centra preserve a birth (neonatal) line^61^: a distinct line separating the inner, trabecular area in two parts, i.e. pre- and post-birth, and gives an approximation of when birth occurred (e.g. Figures 2A5,A8, 2B6,B8, 3E). Importantly, similar birth lines are visible in published pictures of juvenile *Stenopterygius*^27^and have been identified in other marine vertebrates, among them phocid seals^18,62^. In *Cymbospondylus*, the smallest centra where this is observed is PMO 230.842 (Fig. 2B6, centrum height 24 mm), which means that the series might include five foetal centra from individuals that died during pregnancy, an interpretation that is supported by the abundant presence of calcified cartilage in these centra (Fig. 4D)^24^. In a larger centrum (PMO 231.220, Fig. 2B8; height 38 mm) and for those larger in size, the outer layer is more organized than in the small centra and there are many signs of remodelling. These features likely represent the slowing down of growth when entering adulthood. In *Grippia*, the features are less clear, however the smallest centrum where remnants of a birth line are present is PMO 250.500 (Fig. 2A5, height 9 mm; Fig. 3E,E1,E2), indicating that larger centra are juvenile and adult specimens.

In addition, some larger (juvenile to adult) centra have growth marks (lines of arrested growth, LAGs) in the compacted parts of the outer layer. In *Grippia*, this is evident e.g. in PMO 230.499 (Fig. 3D, height 9 mm), and in *Cymbospondylus* PMO 229.743 (Fig. 4C, height 50 mm) a group of two or three LAGs are present, whereas in PMO 229.741 (height 57 mm), five LAGs occur. The presence of these periodic interruptions in growth indicates episodic growth in both taxa.

Rapid growth is indicated by the presence of woven-parallel (fibrolamellar) complex i.e., the combination of woven-fibered tissue (matrix with large and randomly oriented osteocyte lacunae, typical of static osteogenesis) and primary osteons, as well as enriched vascularization, only present in the outer layer and oriented longitudinally to the main axis for both taxa^63,63–66,68^. The *Grippia* centra have some sparse patches of woven-fibered matrix, associated with globular osteocyte lacunae, in trabeculae in the dorsal portion and in the ventral outer layer in some centra (Fig. 3C), while the main bulk of the vertebra is made of parallel-fibered complex (see Supplementary Results). Conversely, in *Cymbospondylus*, woven-parallel complex is consistently observed in both small and large centra (Fig. 4E,F).

In addition to histology, *Grippia* and *Cymbospondylus* centra display notable differences in microanatomical features (Fig. 2A,B), that reflect locomotion. Locomotion is the major function of the vertebral column in marine tetrapods^69,69,71^ and swimming patterns are reflected in the inner microanatomy and external morphology of the centra^17,21,22^. In sagittal section the *Grippia* centra display a narrower, outer endochondral region^27^ and a wider, inner periosteal region with irregularly arranged trabeculae (Fig. 3B). In contrast, in *Cymbospondylus*, trabeculae are anteroposteriorly aligned (Fig. 4B).

In transverse section, both taxa show relatively cancellous centra throughout, and the centre of growth is located around the notochordal canal. The centrum microanatomy (Figs. 3A and 4A,G) is divided into (1) The notochordal ring surrounding the notochordal canal, with compacted bone; (2) the dorsocentral region supporting the neural canal (3) Paired dorsolateral cones supporting the neural arch, which in *Cymbospondylus* are more distinctive and cover a larger area, extending to the rib articulations. (4) The inner cancellous region is periosteal in origin. Its thickness varies depending on the extent of the cones. (5) The outer bone layer, which varies in thickness.

Overall bone compactness and the extent of the outer bone layer are main differences between the two genera. As statistical measures, calculated parametrs were Compactness versus dorsoventral height (as a proxy for centrum size) and Mean outer layer thickness versus total area. To investigate the relationships, linear regressions (GLM) were conducted in PAST v4.13^72^ (Fig. 2D) (see also Supplementary results).

*Grippia* has the same overall microanatomy as found in other grippioids, with uneven trabeculae and relatively large intertrabecular spaces^27^. However, *Grippia* centra studied here differ in important aspects from these other grippioids: The larger centra possess a three-layered structure (Figs. 2A and 3A) i. e. a compacted notochordal ring, inner periosteal region with large intertrabecular spaces and lastly, throughout ontogeny, a well-defined compacted outer layer. This outer layer is relatively thicker as compared to that in *Cymbospondylus* (Figs. 2B,D and 4A) and what was previously known for any ichthyopterygian^27,73^. For *Grippia*, the thickness of the outer layer remains similar compared to the total area of the centra, regardless of centrum size (Fig. 2D1). By contrast, in *Cymbospondylus*, the outer layer is thin and becomes relatively thinner compared to centrum area through ontogeny (Fig. 2D3).

In *Grippia*, relative bone compactness also remains similar throughout ontogeny (Fig. 2D2). In contrast, in *Cymbospondylus*, overall compactness decreases with size (Fig. 2D4), which means that in *Cymbospondylus*, vertebral centra became less compact through ontogeny. In contrast to *Grippia*, *Cymbospondylus* has a microanatomy more typical for a pelagic amniote and, like modern whales, shows a more cancellous structure, increased tightness and a virtually non-existent compact outer layer (Figs. 2B and 4A). Its microanatomy is more consistent in larger specimens: dorsolateral cones with unorganized trabeculae, and a distinct circumferential organisation of the trabeculae in most of the periosteal region, corresponding to the trabecular organisation in the dorsocentral region (Fig. 4A).

## Discussion

### Bone microstructure implications for ontogeny

The presence of woven-parallel (fibrolamellar) complex implies rapid skeletal growth^64,65^ and is present in patches in *Grippia* centra and more consistently in *Cymbospondylus*. Woven-parallel complexes have been reported in Early Triassic *Omphalosaurus* and *Utatsusaurus*, Middle Triassic *Mixosaurus* and *Cymbospondylus*, as well as the Jurassic, parvipelvian taxa *Stenopterygius* and *Ichthyosaurus*^14,27,43,74,75^. Therefore, it appears to be a general ichthyopterygian feature. However, our data demonstrate that the rate of this growth changed during the transition from ichthyopterygian to ichthyosaurs, with *Cymbospondylus* and later ichthyosaurs having higher growth rates as compared to *Grippia*.

*Cymbospondylus* also displays circumferential trabeculae (Figs. 2B10–15, 4H) combined with cones with contrasting growth. Interestingly, a similar microanatomical organisation as in *Cymbospondylus* has been linked to rapid growth in temnospondyls and placodont sauropterygians^23,65^. It is also observed in some early whales^76^. Circumferential organisation of trabeculae is also observed in some ichthyosaurs from the Middle Triassic onwards^27^. Other features, such as the dorsolateral cones of *Cymbospondylus* (Fig. 4A) likely result from the influence of articulating skeletal elements, as they were situated adjacent to the neural arch and rib articulations and might reflect structural stress.

The bone compactness of *Cymbospondylus* decreases through ontogeny (47.3–27.6%; Fig. 2D), whereas this is not the case in *Grippia*. Larger differences in compactness have been associated with ecological differences in a study on plesiosaurs^77^; however the sampled elements may not have been conspecific^16^and the habitat differentiation is not the case for all plesiosaurs^78^. Here, we included several centra of varying sizes from the same area, and they do not indicate habitat partitioning between age groups, similar to hydropelvic mosasaurs, where microanatomy is adapted to the same environment through ontogeny^24^.

### Bone microstructure implications for locomotion

Microstructural differences between *Grippia* and *Cymbospondylus*, and a tandem shift in anatomy and microanatomy, has been noted before, but not attributed to swimming style and rather to evolution of disk-like centrum shapes^27^. However, we interpret the data from the ontogenetic series to clearly indicate differences in locomotion, making it possible to pinpoint the transition from axial undulation to caudal oscillation^79^. In *Grippia*, dorsal vertebrae were relatively anteroposteriorly long and spool-shaped^32,37,80^. This provided a greater range of motion^81^and much of the vertebral column was likely used in anguilliform swimming, which also involved a long tail as compared to the trunk length^33,81,81,82,84^. Conversely, *Cymbospondylus* had many dorsal vertebrae that were anteroposteriorly short^7,9,39^stiffening the trunk^81^with propulsion driven mainly by tail movements. Among modern dolphins and porpoises with analogous body form, longer centra are associated with coastal areas versus open ocean taxa^82^. Based on the differences between *Grippia* as compared to ichthyosaurs such as *Cymbospondylus*^27^the same evolutionary trajectory and ecological diversification seem to have taken place rapidly among early ichthyopterygians.

Ecological differentiation between taxa is further underlined by the consistent and remarkable differences in the vertebral microanatomy and histology between these two taxa. Microanatomically, *Cymbospondylus* shares an overall cancellous inner structure and a thin outermost layer with many pelagic amniotes, whereas *Grippia* has a relatively thicker compacted outermost layer throughout ontogeny, and together with other features of its vertebrae it does not resemble those of any other vertebrate. However, this outer layer is far from approaching the thickness observed in many perhaps more coastal early sauropterygians, corresponding to bone mass increase^19^ or to that evident in the early cetacean *Basilosaurus*^76^. The compacted outer layer of *Grippia* to some extent resembles that of the manatee *Trichechus*^22^ and the mosasaur *Dallasaurus*^23^. Previous studies have not found a direct relationship between the thickness of an outer layer of vertebrae and habitat, and among semi-aquatic amniotes, some have a thickened outer layer whereas other do not^22^. Except for *Basilosaurus* and *Remingtonosaurus*, other early, fully aquatic cetaceans have cancellous vertebrae without a compact outer layer^76,85^.

The bone compactness in *Grippia* centra varies from 46.0 to 61.1% (Fig. 2D2) and has no significant relationship to centrum size. This result contrasts with previous statements that all ichthyopterygian vertebrae had < 50% compactness^73^and confirms the possibility of higher compactness indicated by Houssaye et al.^27^.

One *Grippia* centrum (PMO 250.498) is clearly an outlier (Fig. 2A7). Its compactness is much higher (84.8%), which is visible in all parts of the centrum in transverse view, except the ventralmost portion. It however conforms to the overall *Grippia* internal organization in terms of the surrounding outer layer, and the increased compactness around the notochordal canal. The reason for the differences is unknown as there is no apparent difference from the other centra in outer morphology, taphonomy or infilling by sediments or minerals, but one possibility is pathology.

Cancellous skeletal elements have evolved repeatedly in pelagic marine tetrapods^27,85,85,86,88^ including ichthyosaurs, therefore its presence in *Cymbospondylus* confirms that it had a microanatomy shared with later ichthyosaurs^27^. The adaptive value of compact and cancellous parts of vertebral centra in marine tetrapods is not fully understood. In whales, increased length of trabecular networks seem to be adaptive, to withstand the mechanical stress from locomotion^17^. Cancellous bone can also be an adaptation to increased lipid storage especially in deep divers^89,89,91^.

The anteroposteriorly directed trabeculae in *Cymbospondylus*, compared to the disorganized trabecular network in *Grippia* (Figs. 3A and 4A) also suggest differing locomotion styles. Trabeculae are commonly oriented in the direction of maximum stress^27^and in a comparative study, the trabecular orientation of coastal and freshwater, slow-moving marine mammals were less anteroposteriorly structured as compared to more pelagic, faster moving taxa^17^. Based on this, our findings suggest that *Cymbospondylus* centra experienced more unidirectional and higher anteroposteriorly directed stress, than *Grippia*, which is consistent with *Grippia* being more anguilliform, whereas *Cymbospondylus* had a stiffened trunk and movement was powered by the tail.

### Implications for ichthyosaur evolution

We interpret the microanatomical differences in the outer layer and the compactness between the two taxa as stemming from differences in ecology. *Grippia* and *Cymbospondylus* inhabited the same seas, but used the habitat in different ways, with different locomotory styles. The former used large parts of its vertebral column involved for moving at slower speeds, and it might have dived in shallower areas. For *Cymbospondylus*, propulsion was more tail-driven, and the vertebral column was stiffened. It probably swam faster, dived deeper, and used the open ocean. By extension, these findings indicate that they used different food sources, which is supported by the substantial difference in body size as well as in tooth shape, size and replacement pattern^92,93^.

These findings are significant since they indicate that pelagic ichthyosaurs evolved as early as the Early Triassic (at least by the Spathian) which has been suggested previously^11^. This supports the hypothesis that complex marine ecosystems with large, open ocean predators rapidly evolved soon after the End Permian Mass Extinction^10,12,13^. Our analyses further suggest that the shift from nearshore to pelagic environments for at least one ichthyosaur lineage happened rapidly in the evolution of ichthyosaurs during the recovery phase of the biggest mass extinction event. Furthermore, our results imply a deeper divergence of the grippidian versus ichthyosaurian morphotypes, and the explosive radiation into different habitats and swimming styles which fostered ecological niche partitioning among ichthyosaurs.

The adaptations observed in *Cymbospondylus* may have facilitated the evolution of giant body size in this genus^7,39,41^especially in combination with rapid ontogenetic growth. Physiological signals indicating endothermy-like physiology and rapid growth challenge previous interpretations which assumed marine reptiles preferred warmer conditions^13^. All the large-sized Early Triassic ichthyosaurs are found in non-Tethys seas^7,39,94,95^ and inherently accelerated growth and high metabolism were likely advantageous. Ichthyosaurs evolved rapid growth and pelagic adaptations soon after the End Permian mass extinction and it is evident that the adaptations discussed above were key to the successful explosive radiation and globalization of ichthyosaurs.

## Methods

### Thin section preparation

This study focuses both on microanatomy and histology, studied from thin sections of the vertebrae. Prior to thin sectioning, CT scans of the *Cymbospondylus* centra were performed, whereas for *Grippia* this was not executed due to a large amount of available material. The scans confirmed that they do not provide the necessary resolution making thin sectioning necessary. Note that the CT scans (that can be 3D printed) limit the damage by destructive sampling. Measurements taken before thin sectioning were: dorsoventral height; height from the middle of the notochordal canal to the dorsal margin; anteroposterior length at diapophysis; and maximum mediolateral width (Supplementary Table 1).

For thin sectioning, the ichthyopterygian vertebrae were impregnated using the epoxy EpoFix Resin using the methodology outlined in Chinsamy and Raath^96^. Some were made at the Department of Geosciences, University of Oslo, some at NGU Trondheim, and the remaining at the Centre de Recherche en Paléontologie, at the Muséum national d’Histoire naturelle, Paris. At the former, CaldoFix was used for attachment to a glass plate. Coarse polishing was done using Logitech and Buehler Phoenix 4000 polishing machines before polishing with a Thorlag grinding and polishing automat. At MNHN Paris, the protocol outlined in Lamm 2013^97^ was followed. All centra were sectioned in the transverse plane as close as possible to the anteroposterior midpoint, and some in addition in the sagittal plane, through the centre of the vertebra. The thickness of the final sections were 50–100 micrometres.

### Microstructure – qualitative analysis

Pictures were taken using a Leica DMLP microscope equipped with a Leica MC H170 HD camera and with the software Leica Application Suite EZ v3.4.0. Pictures were taken at various magnification using linear polarized light (LPL), cross polarized light (XPL), and XPL associated with a lambda wave plate (XPL + 1/4λ). Thin sections were scanned with a Nikon Super Coolscan 4000 using the VueScan64 software (9 × 64, version 9.7.02 Professional edition, hamrick.com). The terminology used is based on Buffrénil and Quilhac 2021^67^. We use the term “outer layer” for the outermost portion of the centra, as this is not a fully compacted layer as might be implied by the term “compacta”. “Cortex” in histological terminology implies a certain growth process, which is also not the case for the part we are interested in here. We use presence or absence of woven-parallel complex as a qualitative proxy for rapid growth^65,66^.

### Microstructure – quantitative analysis

Using scans of the thin sections, black and white pictures were obtained with the “adjust threshold” function in ImageJ. The following measurements were taken for quantitative analyses: Total area using ImageJ v1.8.0_345^98^; Compactness: ImageJ v1.8.0_345^98^ on black and white pictures; Outer layer thickness. The latter was calculated from the mean of measurements from five sites, except the dorsal margin, because of the neural arch facets. One *Cymbospondylus* specimen (PMO 229.745) was too incomplete for total area or outer layer thickness to be measured but is included in the analysis of compactness versus dorsoventral height. Based on the other centra, dorsoventral height for this specimen is estimated to be 74 mm.

All measurements were log transformed prior to analysis. Calculated parametres were Compactness versus dorsoventral height and mean cortical thickness versus total area. For comparing compactness, dorsoventral height was used as a proxy for size.

To investigate whether the parametres are related to size only, centra from the two genera with the same dorsoventral height were compared and the absolute thickness of the outer layer compared. *Grippia* PMO 250.501 and *Cymbospondylus* PMO 230.686 both have a height of 7 mm, whereas their outer layer thickness are 0.41 mm and 0.18 mm, respectively. *Grippia* PMO 250.496 and *Cymbospondylus* PMO 231.342 both have a height of 11 mm, whereas their outer layer thickness is 0.62 mm and 0.21 mm, respectively.

## Supplementary Information

Below is the link to the electronic supplementary material.

Supplementary Material 1

## Data Availability

All data generated or analysed during this study are included in the supplementary information files.

## Museum abbreviation

PMO: Natural History Museum, University of Oslo, palaeontological collection.
