# Supplementary material for: Vertebral microstructure marks the emergence of pelagic ichthyosaurs soon after the End Permian Mass Extinction
Source: Sci Rep. 2025 Sep 5;15:30221. doi: 10.1038/s41598-025-14335-y (PMC12413447; doi:10.1038/s41598-025-14335-y)
Supplement: Supplementary file 1 — Supplementary Material 1 [file 41598_2025_14335_MOESM1_ESM.pdf]

**Delsett et al.**

# Supplementary table and text

## Materials

| Taxon | Museum number | Height dorsoventral (mm) | Length anteroposterior (mm) | Total area ($mm^2$) | Compactness % | Outer layer thickness mm | Stratigraphy |
|---|---|---|---|---|---|---|---|
| *Grippia* sp. | PMO 250.504 | 4 | 4 | 12.8 | 58.2 | 0.2 | GBB |
| *Grippia* sp. | PMO 250.503 | 5 | 5 | 13.6 | 49.2 | 0.2 | GBB |
| *Grippia* sp. | PMO 250.502 | 5.5 | 6 | 32.1 | 46.5 | 0.3 | GBB |
| *Grippia* sp. | PMO 250.501 | 7 | 7 | 36.4 | 46.0 | 0.4 | GBB |
| *Grippia* sp. | PMO 250.499 | 9 | 8 | 71.9 | 53.4 | 0.4 | GBB |
| *Grippia* sp. | PMO 250.500 | 9 | 8 | 58.5 | 51.9 | 0.6 | GBB |
| *Grippia* sp. | PMO 250.497 | 10 | 10 | 70.1 | 55.7 | 0.5 | GBB |
| *Grippia* sp. | PMO 250.498 | 10 | 10 | 90.0 | 84.8 | 0.7 | GBB |
| *Grippia* sp. | PMO 250.496 | 11 | 11 | 94.1 | 60.3 | 0.6 | GBB |
| *Grippia* sp. | PMO 233.890 | 16 | 13 | 173.9 | 61.1 | 0.7 | GBB |
| *Cymbospondylus* sp. | PMO 229.744 | 6 | NA | 27.4 | 47.3 | 0.1 | LSN |
| *Cymbospondylus* sp. | PMO 230.686 | 7 | 4 | 42.6 | 69.3 | 0.2 | LSN |
| *Cymbospondylus* sp. | PMO 231.342 | 11 | 6 | 109.5 | 52.7 | 0.2 | LSN |
| *Cymbospondylus* sp. | PMO 230.738 | 16 | 9 | 220.7 | 54.7 | 0.3 | LSN |
| *Cymbospondylus* sp. | PMO 231.337 | 18 | 10 | 147.5 | 44.4 | 0.5 | LSN |
| *Cymbospondylus* sp. | PMO 230.842 | 24 | 13 | 617.9 | 58.8 | 0.4 | LSN |
| *Cymbospondylus* sp. | PMO 230.870 | 36 | NA | 1331.9 | 33.9 | 0.4 | LSN |
| *Cymbospondylus* sp. | PMO 231.220 | 38 | NA | 659.5 | 37.7 | 0.6 | LSN |
| *Cymbospondylus* sp. | PMO 230.627 | 42 | 20 | 1469.1 | 35.0 | 0.6 | LSN |
| *Cymbospondylus* sp. | PMO 229.734 | 43 | 24 | 1673.5 | 37.3 | 0.3 | LSN |
| *Cymbospondylus* sp. | PMO 229.743 | 50 | 24 | 1687.6 | 42.6 | 0.5 | LSN |
| *Cymbospondylus* sp. | PMO 229.740 | 55 | 26 | 2558.6 | 39.3 | 0.5 | LSN |
| *Cymbospondylus* sp. | PMO 229.741 | 57 | 27 | 1266.9 | 32.4 | 0.3 | LSN |
| *Cymbospondylus* sp. | PMO 229.735 | 66 | 30 | 3029.1 | 34.9 | 0.5 | LSN |
| *Cymbospondylus* sp. | PMO 229.745 | NA | 30 | NA | 27.6 | NA | LSN |

**Supplementary table 1.** The sampled specimens and measurements.

### Stratigraphical age

All *Grippia* specimens originate from the Grippia bonebed (GBB). The Grippia bonebed is situated in the Vendomdalen Member, Vikinghøgda Formation [1,2] in the *Pechorosporites disertus* composite Assemblage Zone [3]. Conodonts date the Grippia bonebed to an early but not earliest Spathian [4] at approximately 249 m.a.

The *Cymbospondylus* specimens originate from the Lower Saurian Niveau (LSN). The Lower Saurian Niveau is found in the uppermost part of the the Vendomdalen Member, Vikinghøgda Formation [1] of Late Spathian age [2,5,6], approximately 247 m.a.

## Results

### Quantitative microanatomy

For *Grippia*, the thickness of the outer layer remains similar compared with the total area of the centra, regardless of centrum size (Fig. 2C1). The slope is 0.5, which means isometry because the x axis is area ($mm^2$). Linear relationship, outer layer vs area $p<0.01$. For *Cymbospondylus* the outer layer vs total area also shows a significant linear relationship (Fig. 2C3, $p<0.01$, slope 0,3).

In *Grippia*, relative bone compactness also remains similar throughout ontogeny (Fig. 2C2); There is no linear relationship in compactness vs height ($p = 0.21$). In contrast, in *Cymbospondylus*, overall compactness decreases with size (Fig. 2), which means that in *Cymbospondylus*, vertebral centra became less compact through life, (compactness vs height has a linear relationship, $p<0.01$).

.

**Qualitative microanatomy and histology**
***Grippia***
All *Grippia* centra share an outer layer of relatively compact bone, with some simple longitudinal vascular canals evenly distributed (Figs. 2A, 3A), even in the smallest centra. The tissue in the outer layer is mainly parallel-fibered bone. Some smaller patches of woven-fibered tissue, associated with globular osteocyte lacunae, can be observed, such as in the lateral wall of the projected cones (Fig. 3C). It has to be noted that, contrary to *Cymbospondylus*, the projected cone in Grippia does not have a conic shape, but we use the same name to underline their putative homologous nature.

The smallest centra (PMO 250.502, PMO 250.503) have calcified cartilage in the notochordal canal, directly connected to the notochordal ring in several places, and with a gradual change from the cartilage to ossified bone tissue (Fig.3E). The notochordal ring itself is mainly composed of compacted trabeculae. The matrix constituting the trabeculae has patches of calcified cartilage (e.g. PMO 250.503, PMO 250.504, PMO 250.500), suggesting endochondral ossification was ongoing. In the smallest centra, trabeculae immediately surrounding the notochordal ring are unorganized, and outside of this, trabeculae form a scaffold connecting to the outer edge of the section (Fig. 2A, 3A). These trabeculae are composed of anisotropic tissue with elongated osteocyte lacunae, like those in the notochordal ring. The same features, which suggest an ongoing endochondral ossification, probably means a common origin with the notochordal ring.

The dorsocentral projection has a narrow outer layer, and in the ventral area, a distinct whitish line (e.g. PMO 250.503), in the second smallest centrum (Fig. 2A2, 3E). The process of osteogenesis seems to change across this line: the outer layer does not display any evidence of endochondral ossification, but a deposition of a dense tissue, punctured by many cavities. Given the discontinuity of the tissue, it seems these cavities are erosion bays, probably formed from primary vascularity expanding in the primary tissue. It is absent in all the largest specimens as this part of the trabecular bone has undergone resorption of the primary tissue deposited through endosteal bone. Some of the larger intertrabecular spaces are also the result of taphonomic damage, especially in the smallest centra. Trabeculae are more homogeneous and much tighter in large specimens.

In the larger specimens (Fig. 2A e.g. PMO 250.500 (Fig. 2A5), PMO 250.497 (Fig. 2A8), PMO 233.890 (Fig. 2A10)), the internal structure gradually changes toward 1) an expansion of the dorsolateral cones in the dorsal direction and 2) an associated expansion of the outer layer from the ventral side, so that it progressively invades all edges of the section with the exception of the dorsal tips of the cones, which remain free of compacta (Fig. 2). However, two different types of the outer layer can be distinguished: the dorsocentral one and the one occupying the rest of the section, from the lateral margin of a cone to the other. The latter type shows evidence of periosteal growth, with some regions with isotropic tissue associated with globular and large lacunae, typical of a woven-parallel complex. In these specimens, the internal portion of the centrum is cancellous, with large intertrabecular spaces and relatively thick trabeculae. In most, they are connecting the compact outer layer to the layer of compact bone surrounding the notochord. The tightness of the spongiosa increases dorsally in the largest specimens.

The density of osteocyte lacunae is high in all centra regardless of size, also in the outer layer. Osteocyte lacunae are large and varying in shape in the periosteal bone. Sharpey’s fibres are observed

in the dorsolateral margin of some centra (e.g. PMO 250.496), but never ventrally. In the tip of the cone projection, the SO osteocytes lacunae are also associated with a bundle of Sharpey's fibres.

***Cymbospondylus***

For all ontogenetic stages, the vertebrae are cancellous throughout, with no real compact bone, and with intertrabecular spaces relatively smaller than in *Grippia* (Fig. 2B). Woven-parallel complex is observed in small and large centra (Fig. 4EF).

In *Cymbospondylus*, all sizes display cancellous bone that is primary and not developed through resorption. In transverse view, the trabeculae are well-organized in the outer area, with increased compactness closer to the outer layer. Trabeculae are increasingly ordered with increasing size of the centrum, as is the vascularization in the outer layer. In the dorsocentral projection, the trabeculae are laid down regularly, whereas in the dorsolateral cones, they are randomly organized. The outer layer is thin and vascularized (Fig. 4A, H). There are many osteocyte lacunae in the cortical layer, varying in size and orientation but all relatively large. As for *Grippia*, the trabeculae are primarily made up of primary bone, with a layer of dense parallel-fibered bone deposited centripetally.

Porous external texture, often used as an osteological indicator of early ontogeny, is observed in the smallest *Cymbospondylus* centra (PMO 229.744 and PMO 231.342). One of the smallest specimens (PMO 231.342, Fig 4D) displays clear signs of endochondral bone ossification: calcified cartilage occupies a large part of the notochordal canal, likely indicating a foetal stage. The vascularization has longitudinally oriented osteons. The tissue is increasingly more compact closer to the bony notochordal ring. It takes the shape of lines of large, rounded cell lacunae in an amorphous tissue, which is typical of calcified cartilage[7], before ossifying and constituting the bony ring. The spongiosa in the smallest vertebrae appear to have been laid down primarily as there are no traces of remodelling or resorption.

In the larger specimens, most evidence of calcified cartilage disappear, and the ventral outer layer progressively expand on the lateral flanks until reaching almost two-thirds of the height of the centrum. In the transition between this and the dorsolateral cones with unorganized trabeculae, Sharpey's fibres at the site of the apophyses are indicative of insertion of muscle or ligaments, probably ligaments for the rib head. While most of the centrum is constituted by disorganized trabeculae, the level of disorganization seems to be higher in the dorsolateral cones, suggesting a more rapid growth. This hypothesis is corroborated by the proportionally more extensive area of these cones in larger compared to smaller centra.


1. Mørk, A., Elvebakk, G., Forsberg, A.W., Hounslow, M.W., Nakrem, H.A., Vigran, J.O., and Weitschat, W. (1999). The type section of the Vikinghøgda Formation: a new Lower Triassic unit in central and eastern Svalbard. Polar Res. *18*, 51-82. doi:10.1111/j.1751-8369.1999.tb00277.x.
2. Hansen, B.B., Hammer, Ø., and Nakrem, H.A. (2018). Stratigraphy and age of the *Grippia* niveau, Lower Triassic Vikinghøgda Formation, Spitsbergen. Norwegian Journal of Geology *98*, 175-187.
3. Vigran, J.O., Mangerud, G., Mørk, A., Worsley, D., and Hochuli, P.A. (2014). Palynology and geology of the Triassic succession of Svalbard and the Barents Sea (Geological Survey of Norway).
4. Nakrem, H.A., and Orchard, M.J. (2023). Conodonts from the Grippia niveau bonebed (Lower Triassic, Spathian), Spitsbergen, Arctic Norway. Lethaia *56*, 1-10. 10.18261/let.56.4.7.
5. Hurum, J.H., Engelschiøn, V.S., Økland, I., Bratvold, J., Ekeheien, C., Roberts, A.J., Delsett, L.L., Hansen, B.B., Mørk, A., Nakrem, H.A., et al. (2018). The history of exploration and

stratigraphy of the early to middle Triassic vertebrate bearing strata of Svalbard (Sassendalen Group, Spitsbergen). Norwegian Journal of Geology *98*, 165-174.
6. Engelschiøn, V.S., Delsett, L.L., Roberts, A.J., and Hurum, J.H. (2018). Large-sized ichthyosaurs from the Lower Saurian niveau of the Vikinghøgda Formation (Early Triassic), Marmierfjellet, Spitsbergen. Norwegian Journal of Geology *98*, 239-266.
7. Quilhac, A. (2021). An overview of cartilage histology. In Vertebrate Skeletal Histology and Paleohistology, V. Buffrénil, A. Ricqlès, L. Zylberberg, and K. Padian, eds. (CRC Press), pp. 123-135.
